# Wheat Gene *TaATG8j* Contributes to Stripe Rust Resistance

**DOI:** 10.3390/ijms19061666

**Published:** 2018-06-05

**Authors:** Md. Abdullah-Al Mamun, Chunlei Tang, Yingchao Sun, Md. Nazrul Islam, Peng Liu, Xiaojie Wang, Zhensheng Kang

**Affiliations:** 1 State Key Laboratory of Crop Stress Biology for Arid Areas and College of Plant Protection, Northwest A&F University, Yangling 712100, China; mamunrwrc@yahoo.com (M.A.-A.M.); tclbad@163.com (C.T.); sycresearch@sina.com (Y.S.); mnislam30@yahoo.com (M.N.I.); Wood319@126.com (P.L.); wangxiaojie@nwsuaf.edu.cn (X.W.); 2 Regional Wheat Research Centre, Bangladesh Agricultural Research Institute, Shyampur, Rajshahi-6212, Bangladesh; 3 Key Laboratory of Integrated Pest Management on the Loess Plateau of Ministry of Agriculture, Northwest A&F University, Yangling 712100, China; 4 Agrochemical and Environmental Research Division, Institute of Food and Radiation Biology, Atomic Energy Research Establishment, Bangladesh Atomic Energy Commission, Ganakbari, Savar, Dhaka-1349, Bangladesh

**Keywords:** *Puccinia striiformis* f. sp. *tritici*, virus-induced gene silencing, *Triticum aestivum*, ATG8, Agrobacterium-mediated transient expression

## Abstract

Autophagy-related 8 (ATG8) protein has been reported to be involved in plant’s innate immune response, but it is not clear whether such genes play a similar role in cereal crops against obligate biotrophic fungal pathogens. Here, we reported an *ATG8* gene from wheat (*Triticum aestivum*), designated *TaATG8j*. This gene has three copies located in chromosomes 2AS, 2BS, and 2DS. The transcriptions of all three copies were upregulated in plants of the wheat cultivar Suwon 11, inoculated with an avirulent race (CYR23) of *Puccinia striiformis* f. sp. *tritici* (*Pst*), the causal fungal pathogen of stripe rust. The transient expression of *TaATG8j* in *Nicotiana benthamiana* showed that TaATG8j proteins were distributed throughout the cytoplasm, but mainly in the nucleus and plasma membrane. The overexpression of *TaATG8j* in *N. benthamiana* slightly delayed the cell death caused by the mouse apoptotic protein BAX (BCL2-associated X protein). However, the expression of *TaATG8j* in yeast (*Schizosaccharomyces pombe*) induced cell death. The virus-induced gene silencing of all *TaATG8j* copies rendered Suwon 11 susceptible to the avirulent *Pst* race CYR23, accompanied by an increased fungal biomass and a decreased necrotic area per infection site. These results indicate that *TaATG8j* contributes to wheat resistance against stripe rust fungus by regulating cell death, providing information for the understanding of the mechanisms of wheat resistance to the stripe rust pathogen.

## 1. Introduction

Plants are subjected to various biotic and abiotic stresses [1]. Upon environmental stimuli, autophagy is upregulated to sense the intracellular stimuli and mount reactions to protect plants from damage [2]. Autophagy, which is also called self-eating, is a catabolic process in eukaryotic cells that cleans up excessive or unwanted cellular components and macromolecules for nutrient recycling [3,4]. Throughout this process, cytoplasmic components, for example, proteins and different organelles, are targeted to either lysosomes/endosomes or vacuoles for degradation inside these compartments [5]. As a highly regulated and dynamic process that degrades dysfunctional or unnecessary cellular components, autophagy plays key roles in modulating plant cell homeostasis. Autophagy has been linked to plants’ defense responses against microbes, promoting either death or survival [6,7,8]. Upon destructive necrotrophic fungal infection, autophagy negatively regulates plant defenses, playing an anti-death role to limit the disease lesion [9]. During biotrophic pathogen interactions, autophagy is a part of the defense response [6,10]. The silencing of the Autophagy-related protein 6 (ATG)-heterologous protein in wheat blocks the broad-spectrum immunity conferred by *Pm21* towards *Blumeria graminis* f. sp. *tritici* (*Bgt*), indicating that autophagy positively regulates the wheat defense response against the powdery mildew fungus [11]. NBR1/Joka2-mediated autophagy plays a positive role in the defense against viral and oomycete pathogens [12]. Thus, autophagy-mediated defense responses affect the dynamics of host–microbe interactions.

ATG protein mediates a degradative process through which damaged or unwanted cellular components are recycled. ATG proteins contain the ubiquitin domain of the Gamma-aminobutyric acid (GABA)-receptor-associate protein (GABARAP), which is a large family of proteins that mediate intracellular membrane trafficking and/or fusion. It contains six predicted tubulin binding sites and three ATG7-binding sites. The mechanisms of autophagy in mammalian and plant cells are evolutionarily conserved [13,14]. To date, more than 35 ATG genes have been recognized in yeast, among which 17 core ATGs are engaged in autophagosome formation [15,16]. The lipidation of ATG8 and its position in the membrane are crucial for autophagic membrane extension [17]. Normally, ATG8 is conjugated to the autophagosomal membrane via phosphatidylethanolamine (PE) lipidation [18]. Under autophagy, ATG8 is processed at the C-terminus by ATG4 to expose glycine. The modified ATG8 protein is then conjugated to PE by ATG3 and ATG7 and assembled into autophagosome-resembling structures that are delivered to vacuoles [19]. ATG8 participates in both cytoplasm-to-vacuole transport and autophagy [20]. In plants, the expanded ATG8 protein family members exhibit different expression under different conditions, which may be responsible for the specificity of targeting autophagosomes [21,22]. In Arabidopsis, ATG8 associates with autophagosomal membranes during autophagy [23], and ATG8-mediated autophagy accumulates upon infection with *Pto* DC3000 (AvrRpm1) [6]. Moreover, the ATG8CL protein is targeted by the *Phytophthora infestans* effector PexRD54 to reroute the autophagosome to the feeding sites for nutrient uptake [24]. It appears that ATG8-mediated autophagy plays a significant role in plant effector-triggered immunity (ETI) and is targeted by pathogens to attack the host defenses.

*Puccinia striiformis* f. sp. *tritici* (*Pst*) is a biotrophic fungal pathogen that feeds on living wheat cells to uptake nutrients [9]. Wheat–*Pst* interactions often take place in a gene-for-gene manner. In resistant wheat cultivars, the avirulence (*AVR*) gene of *Pst* is recognized by a corresponding plant resistance (*R*) gene, which subsequently activates rapid cell death surrounding the infection sites, known as the hypersensitive response (HR), to limit pathogen growth and spread [25]. Simultaneously, a set of defense reactions, as well as rapid cell death, cell wall strengthening, pathogenesis-related (PR) proteins, and antimicrobial compounds, are also observed [26]. As a typical feature of plant ETI, understanding the HR mechanism will provide new insights into Avr-R-mediated resistance.

Despite the large number of studies on ATG8 in yeasts and animals, little is known for plants, especially modern bread wheat (*Triticum aestivum*) [27,28]. In this study, a wheat ATG8-heterologous protein, *TaATG8j*, was isolated from wheat cultivar cv. Suwon 11 (Su11) infected with *Pst*. The expression pattern and subcellular localization of *TaATG8j* were investigated. To analyze its role in cell death regulation, *TaATG8j* was heterologously expressed in *Nicotiana benthamiana* and *Schizosaccharomyces pombe*. Furthermore, transient silencing of *TaATG8j* expression in wheat was performed to analyze the function of *TaATG8j* in wheat resistance to *Pst*.

## 2. Results

### 2.1. Identification and Isolation of the TaATG8j Gene

Based on the expressed sequence tag (EST) sequence (GenBank Accession No. GR302394.1) from the cDNA library of a compatible wheat-*Pst* interaction, a 695-bp cDNA sequence with a 360-bp open reading frame(ORF) was obtained from Su11 wheat, which encoded an ATG8-heterologous protein. A BLASTN (Basis Local Alignment Search Tool Neuclitide) search in the Chinese Spring genomic database revealed three copies of this gene in the wheat genome (Figure S1), located on the wheat chromosomes 2AS, 2BS and 2DS. To obtain more information about *TaATG8j* in the wheat sub-genomes, we downloaded the exon–intron structure and assembled exon sequences, designated *TaATG8j-2AS*, *TaATG8j-2BS* and *TaATG8j-2DS*, respectively (Figure S1 and Table 1). The *TaATG8j* gene from Su11 shared 99.72%, 98.61% and 98.34% nucleotide identity with the three copies of *TaATG8j-2AS*, *TaATG8j-2BS* and *TaATG8j-2DS* of Chinese Spring in the ORF region (Figure S1). However, considering the protein sequence, only one amino acid variation was observed in TaATG8j, with the predicted proteins of the three homoeologous genes (Figure S2). Considering exon structure, all three copies of *TaATG8j* consist same number(five) of exon (Figure S3).

**Figure 1:**
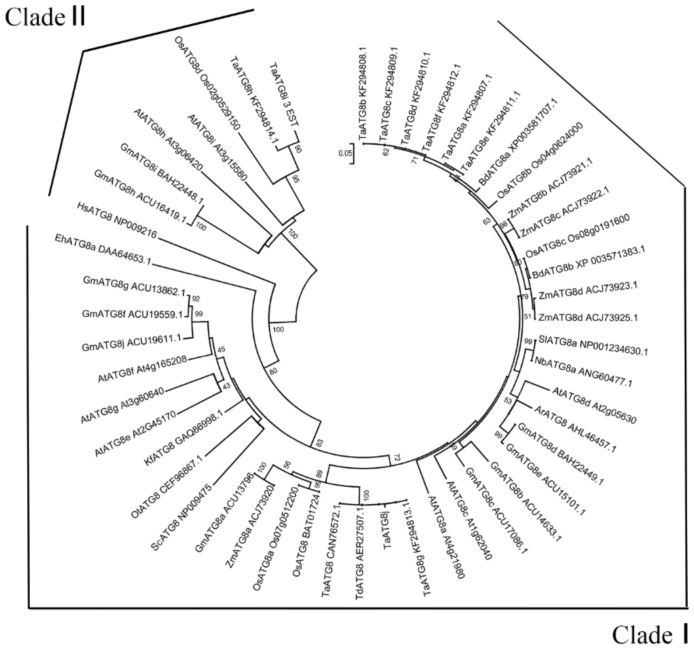
Phylogenetic analysis of *TaATG8j* with other members of ATG8 (autophagy-related 8) family. The phylogenetic tree was constructed using the neighbor-joining method and the software MEGA 7. The internal branches were assessed by bootstrapping values, with 1000 bootstrap replicates, and a branching cut-off of 50%. The branches are considered protein name, GenBank accession number, and one assembled sequence from three EST (expressed sequence tag) sequences (HX189738, CK196170 and HX250137). Ta: *Triticum aestivum*; Bd: *Brachypodium distachyon*; Os: *Oryza sativa*; Zm: *Zea mays*; Gm: *Glycine max*; At: *Arabidopsis thaliana*; Td: *Triticum dicoccoides*; Sl: *Solanum lycopersicum*; Nb: *Nicotiana benthamiana*; Kf: *Klebsormidium flaccidum*; Sc: *Saccharomyces cerevisiae*; Ot: *Ostreococcus tauri*; Eh: *Emiliania huxleyi*; Hs: *Homo sapiens*.

**Figure 2:**
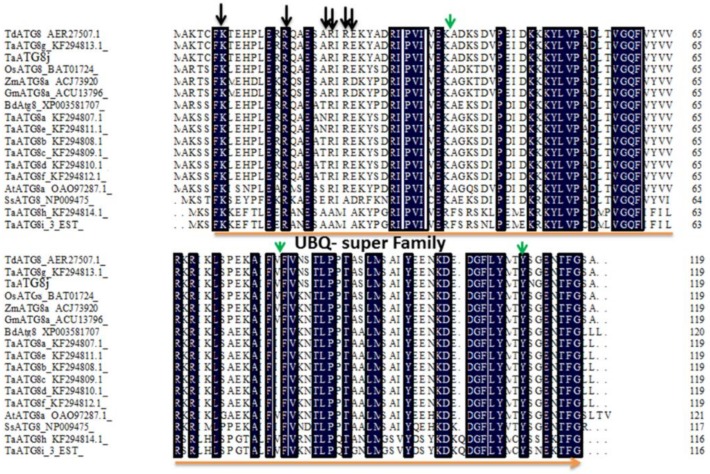
Multiprotein sequence alignment of *TaATG8j* against other members of the ATG8 family. Identical amino acids are indicated in black. Black and green arrows indicate tubulin- and ATG7-binding sites, respectively. Orange line indicates the upiquitin-like (UBQ) super family protein. Ta: *Triticum aestivum*; Td: *Triticum dicoccoides*; Zm: *Zea mays*; Os: *Oryza sativa*; Gm: *Glycine max*; Bd: *Brachypodium distachyon*; Sc: *Saccharomyces cerevisiae*; and At: *Arabidopsis thaliana*.

Plants contain expanded ATG8 family members. Similarly, here, in addition to the *TaATG8j* gene, we identified nine other ATG8-heterologous proteins in the wheat genome, named *TaATG8a*–*h* (GenBank accession numbers KF294807–KF294814), was along with a heterologous protein assembled from 3EST (HX189738, CK196170 and HX250137) sequences [29], designated *TaATG8i*. Therefore, the ATG8 gene identified in this study was named *TaATG8j* (Figure 1 and Figure 2). A structural sequence analysis showed that *TaATG8j* encoded a putative protein of 119 amino acids, with a conserved UBQ (Upiquitin-like) superfamily domain, six (black arrow) predicted tubulin binding sites and three (green arrow) predicted ATG7 binding sites (Figure 2). Multiprotein sequence alignment with other organisms showed that *TaATG8j* shared the highest identity (95.93%) with *ATG8g* from *T. aestivum* and *ATG8* from *Triticum dicoccoides*. Additionally, *TaATG8j* shared 82.93%, 82.11%, 90.24%, and 86.18% identity with *TaATG8a*–*f* from wheat *BdATG8* from *Brachypodium distachyon*, *OsATG8* from rice and *ZmATG8a* from maize respectively. The lowest identity (less than 70%) was observed for *SsATG8* from yeast and *TaATG8h-i* from wheat (Figure 2).

Phylogenetic analyses indicated two distinct clades that comprised all members of the ATG8 subfamily. Clade 1 comprised plant members including TaATG8j protein, and Clade 2 was related to human ancestors. Most of the paralogs of *Oryza sativa* (Os), *Glycine max* (Gm), *Arabidopsis thaliana* (At) and *T. aestivum* (Ta) were in Clade 1, but some of their paralogs were also present in Clade 2, indicating that the ancestor of Clade 1 and its descendants frequently duplicated during genomic evolution (Figure 1). The lengths of the branches indicate the closeness of the evolutionary relationships between the ATG8 proteins.

### 2.2. TaATG8j Is Induced upon Incompatible Pst Attack

**Figure 3:**
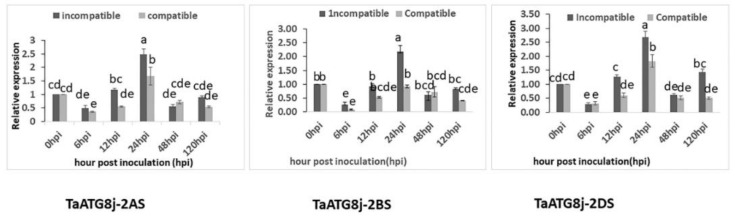
Relative expression levels of the three *TaATG8j* copies in wheat plants (cv. Suwon 11) inoculated separately with incompatible and compatible pathotypes of *Puccinia striiformis* f. sp. *tritici*. The wheat seedlings were inoculated with CYR23 (incompatible) and CYR31 (compatible) and sampled at 0, 6, 12, 24, 48 and 120 hpi. The data were standardized to the wheat elongation factor *TaEF-1α* gene. The relative expression level of the gene was quantified using the comparative threshold (2^−ΔΔ*C*t^) method. Error bars indicate the variation among three replications, and the different letters represent significant differences (*p* < 0.05) by the Tukey HSD (Honestly Significant Difference) test. hpi: hours post-inoculation.

To explore the function of *TaATG8j* in wheat defense to *Pst*, the transcript level of *TaATG8j* was quantified in both compatible and incompatible wheat–*Pst* interactions using different genome-specific primers (Table S1). In the incompatible interaction, the transcripts of all copies of *TaATG8j* were significantly upregulated (more than 2.0-fold) at 24 hpi compared with the control (0 hpi), although the upregulation level differed slightly. In contrast, the compatible interaction suppressed these copies (Figure 3). These results indicate that all three copies (*TaATG8j-2AS*, *TaATG8j-2BS* and *TaATG8j-2DS*) of *TaATG8j* were upregulated in the early stage of the incompatible interaction (24 hpi).

### 2.3. Protein TaATG8j Is Distributed throughout the Cytoplasm but Mainly in Nuclei and Plasma Membranes

**Figure 4:**
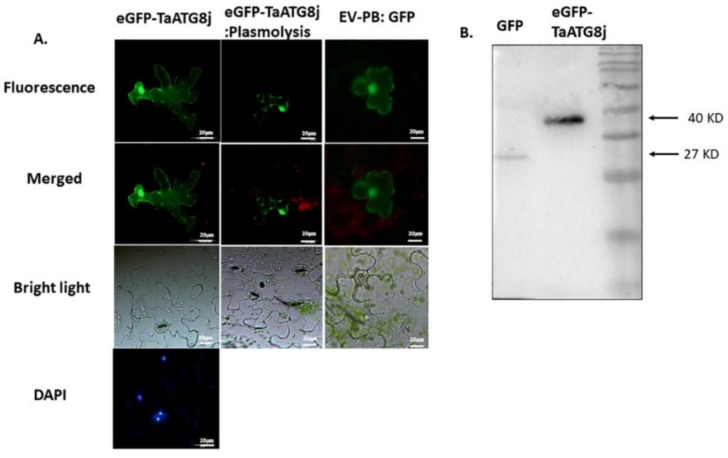
Subcellular localization and immunoblotting of the TaATG8j protein. (**A**) *N. benthamiana* leaf tissues were transformed with a plasmid carrying a fusion protein. The picture was obtained using a fluorescence microscope. The left four images indicate eGFP-TaATG8j, the middle three images indicate eGFP-TaATG8j: Plasmolysis, and the right three images indicate EV-PB: GFP (control); (**B**) Immunoblotting analysis of eGFP-TaATG8j (40 kDa) and EV-GFP (27 kDa). Bar: 20 μm.

The localization of ATG8j proteins on the surface of membranes is necessary for autophagy biogenesis [29]. To determine the subcellular localization of TaATG8j in the leaf tissue of *N. benthamiana*, recombinant PB-eGFP-TaATG8j was infiltrated into *N. benthamiana*. The empty vector (EV) P^BinGFP2^-GFP was used as a control. Using DAPI (4′,6-diamidino-2-phenylindole) and a hypertonic solution, fluorescence microscopy revealed that GFP-TaATG8j fusion proteins were distributed throughout the cytoplasm, especially the nucleus and plasma membrane (Figure 4A). To confirm this result, we further conducted Western blot to analyze the stability of the TaATG8j fusion protein. We successfully observed stable TaATG8j fusion proteins and EV-P^BinGFP2^-GFP proteins at 40 and 27 kDa, respectively (Figure 4B).

### 2.4. TaATG8j Delays Cell Death Triggered by BAX in N. benthamiana

**Figure 5:**
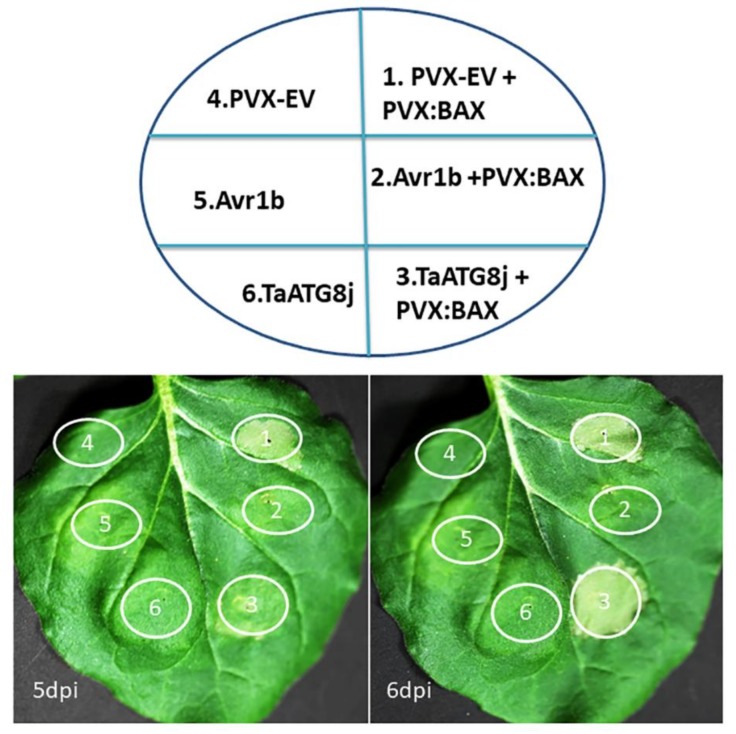
The transient overexpression of *TaATG8j* in *N. benthamiana* (*Nicotiana benthamiana*). Transient overexpression of *TaATG8j* suppressed the programmed cell death induced by the *PVX-BAX* gene. The *N. benthamiana* leaf was infiltrated with *Agrobacterium tumefaciens* cells carrying *TaATG8j* (circles 3 and 6), *Avr1b* (circles 2 and 5) or *EV* (circles 1 and 4). Photographs were taken at 5 and 6 days after the second infiltration (the second infiltration was done only in circles 1, 2 and 3 at 24 h after the first infiltration using *A. tumefaciens* carrying *PVX:BAX*). The circular areas indicate the infiltrated spaces.

To investigate the potential role of *TaATG8j* in Programmed cell death (PCD), we overexpressed *TaATG8j* in *N. benthamiana* through the *A. tumefaciens* (GV3101)-mediated infiltration assay. EV and Avr1b were used as negative and positive controls, respectively. When expressed alone, EV, Avr1b or *TaATG8j* did not cause cell death (Figure 5). Five days post-infiltration with *A. tumefaciens* carrying PVX-BAX, obvious cell death was observed in the sites infiltrated with EV, suggesting its cell induction role. When co-expressed with Avr1b, BAX did not cause cell death, indicating that it was suppressed by Avr1b. For *TaATG8j*, slight cell death was detected at five days after BAX infiltration, while significant cell death was observed at six days post infiltration (Figure 5). These results indicated that *TaATG8j* alone was not able to induce cell death but rather delayed the cell death triggered by BAX.

### 2.5. Overexpression of TaATG8j Induces Cell Death in Yeast

**Figure 6:**
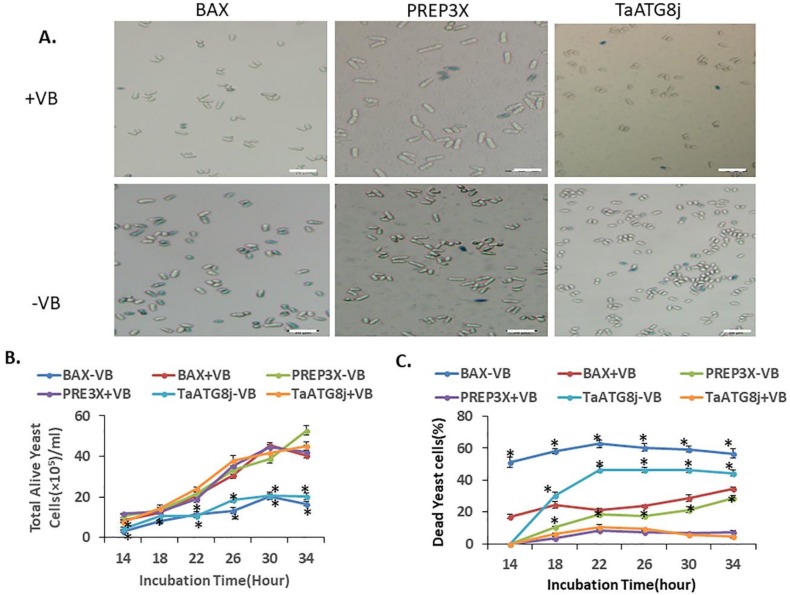
Overexpression of *TaATG8j* in yeast cells. The mouse pro-apoptotic BAX gene and pREP3X empty vector were used as positive and negative controls, respectively. Thiamine (VB) and trypan blue were used for repression and as a staining medium, respectively. The yeast cells were counted using a hemocytometer. Three biological replicates were performed. The yeast cell number mL^−1^ was calculated at 14, 18, 22, 26, 30 and 34 h post-incubation. Incubation was started from an identical OD_600_: 0.2 using ±VB. (**A**) Phenotypically dead and alive yeast cells were compared by staining trypan blue staining using ±VB after expressing pREP3X_TaATG8j, pREP3X_BAX, or pREP3X. The dead yeast cells were stained blue. Bar: 20 µm; (**B**) The total number of cells mL^−1^ was counted. Yeast cells expressing the total number of alive and dead yeast cells of *TaATG8j* and *BAX* were significantly altered compared to the control; (**C**) The percentage (%) of the dead yeast cells of the total cells was calculated. Yeast cells expressing in *TaATG8j* or BAX showed more cell death compared with the control. Mean data are presented, and error bars indicate the variations among the biological replicates. Asterisks indicate the level (* *p* < 0.01) of significance without thiamine (VB) using Student’s *t*-test.

After overexpression in *N. benthamiana* cells, we further investigated the roles of *TaATG8j* in cell death using a fission yeast system. *TaATG8j* was overexpressed in fission yeast (*S. pombe*) governed by the nmt promoter, which is suppressed by thiamine (VB). The mouse *BAX* gene, a pro-apoptotic factor that induces cell death in yeast, and empty pREP3X vector were used as positive and negative controls, respectively [30]. Yeast cells were cultured with (+VB) or without thiamine (−VB), and the cell death phenotype was checked with trypan blue. As shown in Figure 6A, the dead yeast cells were stained blue. The expression of *TaATG8j* or *BAX* led to significantly decreased total alive yeast cells in the absence of thiamine (−VB) compared to that with thiamine (+VB) throughout the incubation period, whereas the expression of pREP3X did not cause any significant change (Figure 6B). Furthermore, the ratio of dead (stained yeast cells) to total yeast cells was calculated. The results showed that yeast cells expressing *BAX* incubated without thiamine (−VB) exhibited significantly more dead cells than those incubated with thiamine (+VB) after 14 to 34 h of incubation (Figure 6C). Similar results were obtained for *TaATG8j*, except at 14 and 18 h after incubation (Figure 6C). However, the yeast cells transformed with empty pREP3X did not exhibit any remarkable change in the ratio of dead cells. These results indicated that the overexpression of *TaATG8j* induced cell death in fission yeast.

### 2.6. Knockdown of TaATG8j Enhances Wheat Susceptibility to Pst

**Figure 7:**
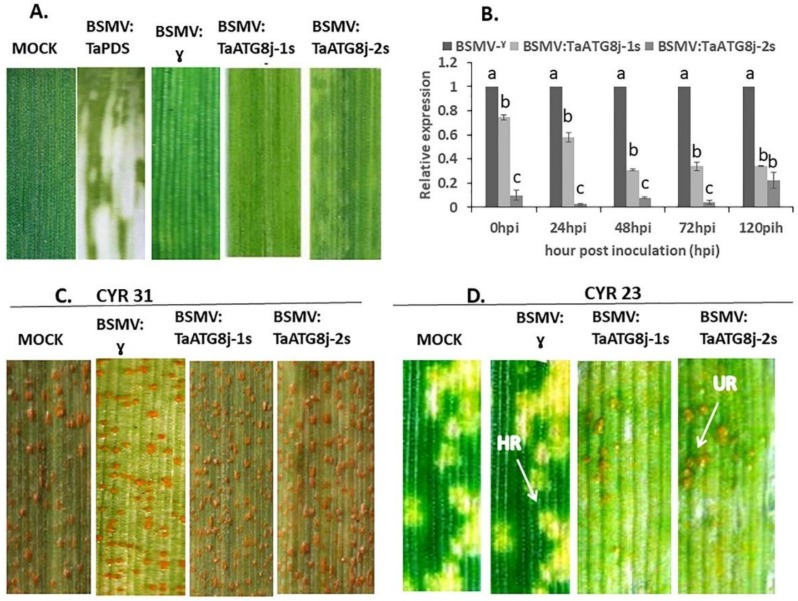
Functional activities of the *TaATG8j* gene mediated by BSMV (Barley Stripe Sosaic Virus) mediated gene silencing during the interaction between wheat and *Puccinia striiformis* f. sp. *tritici*. Wheat seedlings were inoculated by recombinant BSMV:TaPDS, BSMV:γ, BSMV:TaATG8j-1s or BSMV:TaATG8j-2s on the second leaves, followed by inoculation with *Pst* pathotype CYR23 (avirulent) or CYR31 (virulent) on the fourth leaves. (**A**) Slight chlorotic mosaic symptoms appeared on the fourth leaves of wheat seedlings in recombinant BSMV:γ, BSMV:TaATG8j-1s and BSMV:TaATG8j-2s at 13 dpi. Mock leaves were treated only with FES buffer; (**B**) Relative transcription levels of *TaATG8j* in silenced plants after inoculation with CYR23. The data were standardized with the wheat elongation factor gene (*TaEF-1α*). Error bars indicate the variation among the three replications, and different letters indicate significant differences (*p* < 0.05) by the Tukey HSD test; (**C**,**D**) Photographs of the fourth leaves at 18 dpi, which were further inoculated by fresh urediniospores of the virulent strain CYR31 and the avirulent strain CYR23.

To study the role of the *TaATG8j* gene in wheat immunity against *Pst* in more detail, barley stripe mosaic virus (BSMV)-mediated gene silencing (VIGS) was used to silence *TaATG8j*. Due to the high similarity between the three copies, the two fragments used for silencing resulted in the silencing of the three copies together (Figure S1). Wheat seedlings inoculated with BSMV:TaATG8j-1s, BSMV:TaATG8j-2s and BSMV:γ exhibited mild chlorotic mosaic symptoms on the third or fourth leaves at 13 dpi, while the seedlings inoculated with BSMV:TaPDS displayed strong photobleaching symptoms (Figure 7A). These results suggest that the BSMV-VIGS system functioned accurately. Fifteen days post-inoculation with the *Pst* pathotype CYR23, apparent HR was observed on the fourth leaves of the BSMV:TaATG8j-knockdown wheat seedlings. Sporadic fungal sporulation was observed around the necrotic spots at 18 dpi (Figure 7D). In contrast, inoculation with the *Pst* pathotype CYR31 resulted in normal disease development with no remarkable change (Figure 7C).

qRT-PCR analyses showed that the expression of both fragments was significantly reduced in *TaATG8j*-knockdown plants compared with control plants (Figure 7). Considering BSMV:γ, the highest silencing efficiency was observed with the BSMV:TaATG8j-2s fragment at all time points (more than 80%), and the BSMV:TaATG8j-1s fragment was also significantly silenced. These results indicated that the *TaATG8j* gene was effectively silenced in the incompatible interaction, and after silencing the *TaATG8j* gene, the resistant wheat cultivar cv. Su11 became susceptible.

### 2.7. Suppression of Defense-Related Genes in TaATG8j-Knockdown Plants

**Figure 8:**
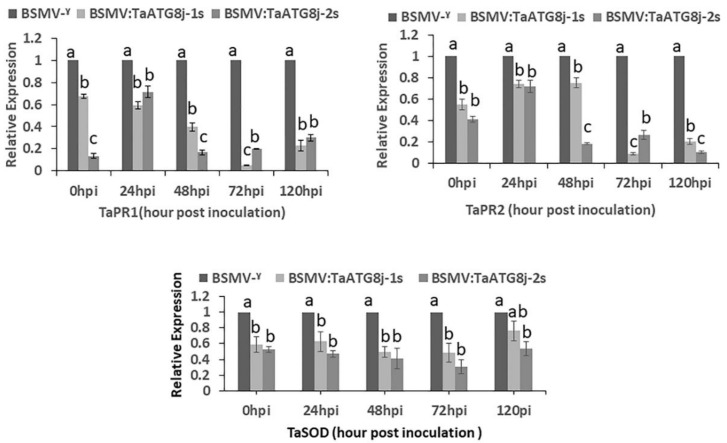
Transcription levels of defense-related genes in response to *Puccinia striiformis* f. sp. *tritici* in *TaATG8j*-silenced plants. The expression of *TaPR1*, *TaPR2* and *TaSOD* was quantified in *TaATG8j*-silenced plants compared with the control. The data were standardized to the wheat *TaEF-1α* gene. Error bars indicate the variations among the three independent biological replicates. Different letters indicate significant differences (*p* < 0.05) using the Tukey HSD test.

At the time of plant pathogenic infection, the production of *PR* proteins in the uninfected parts of the plant can prevent the affected plant from further infection [31,32]. Upon the reduced resistance of wheat Su11 to the avirulent *Pst* CYR23, we further assessed the expression pattern of defense-related genes in *TaATG8j*-knockdown plants by qRT-PCR. The results showed that the *PR* protein genes, *TaPR1* and *TaPR2*, were down-regulated in *TaATG8j-1s*- and *TaATG8j-2s*-knockdown plants infected by *Pst* CYR23, particularly at 48, 72 and 120 hpi (Figure 8 and Figure S4). The expression of the *PR* protein gene *TaSOD* was also reduced in the *TaATG8j*-knockdown plants. This result indicates that the accumulation of defense-related genes was suppressed in *TaATG8j*-knockdown plants.

### 2.8. Histological Observation of Pst Growth and Host Necrotic Cell Death

**Figure 9:**
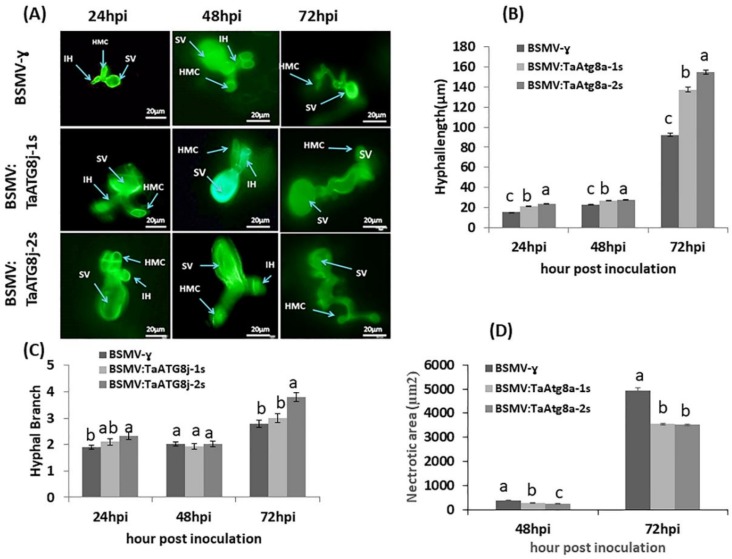
Histological observation of *TaATG8j-*silenced plants challenged with the avirulent *Pst* pathotype CYR23. After removing chlorophylls, the samples were stained with wheat germ agglutinin (WGA). Sample leaves were considered recombinant BSMV:γ, BSMV:TaATG8j-1s and BSMV:TaATG8j-2s that had been previously inoculated with pathotype CYR23. (**A**) Leaves were observed at 24, 48 and 72 hpi. The infected sites were observed using an epifluorescence microscope. HMC: haustorial mother cell; SV: substomatal vesicle; IH: infection hyphae. Each result was considered from 50 infection sites. Bar, 20 µm; (**B**) Hyphal length: the distance from the joint of the substomatal vesicle to the apex of the hypha; (**C**) Hyphal branches: the average numbers of primary hyphae; (**D**) Necrotic area: quantified as the area of autofluorescence. Different letters indicate significant differences (*p* < 0.05) using the Tukey HSD test.

To illustrate the fungal development in *TaATG8j*-knockdown plants, WGA staining was used to stain the fungal structure. As shown in Figure 9A,B, the *TaATG8j*-knockdown plants inoculated with CYR23 had significantly longer hyphae than the BSMV:γ plants at 24, 48 and 72 hpi. In addition, the number of branches was significantly increased (*p* < 0.05) by the second knockdown fragment at 24 and 72 hpi, and more hyphal branches were observed than in the BSMV:γ-infected seedlings (Figure 9C). In contrast, the necrotic areas were significantly decreased in first- and second-fragment infected leaves, followed by BSMV:γ (Figure 9D). The overall histological results indicated that silencing *TaATG8j* enhanced the susceptibility of the Su11 cultivar to *Pst* and permitted enhanced hyphal growth and branching.

### 2.9. Increased Fungal Biomass in TaATG8j-Knockdown Plants

**Figure 10:**
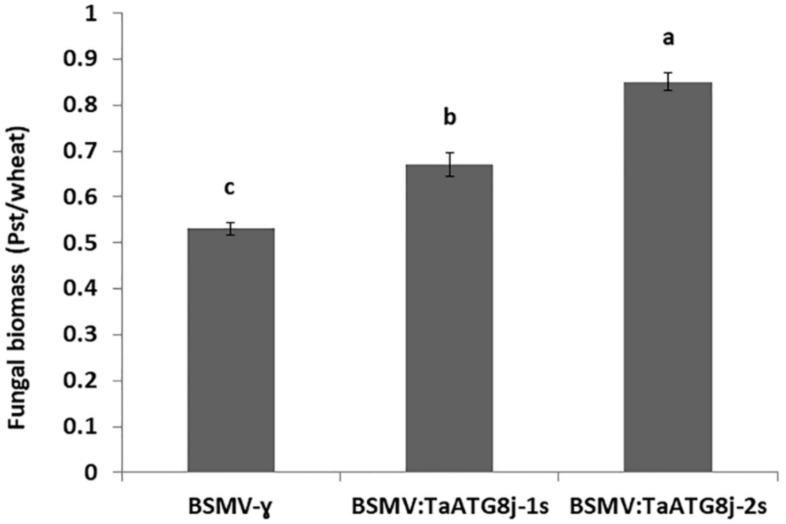
DNA biomass assay in *TaATG8j*-silenced plants challenged with the avirulent *Pst* pathotype CYR23. Two standard curves were generated for the accurate measurement of *Pst* and wheat DNA. Quantification cycle (Cq) values are plotted against the initial copy number of template DNA (10^4^, 10^5^, 10^6^, 10^7^, 10^8^, and 10^9^). Genomic DNA of Su11, infected with urediniospore of *Pst* pathotype CYR23, was used to generate the standard curves. Fungal biomass was measured by qRT-PCR on the extracted total genomic DNA from wheat leaves infected with CYR23 at 18 dpi. The ratio of the total fungal to total wheat DNA was calculated using the *TaEF-1α* and *Pst-EF* genes for normalization. The letters a, b, c indicates the significant difference using LSD test among biomass in BSMV:γ, BSMV:TaATG8j-1s and BSMV:TaATG8j-2s.

To assess the fungal mycelium growth in *TaATG8j*-silenced plants, total genomic DNA was extracted to quantify the fungal biomass using qRT-PCR. At 18 dpi, in *TaATG8j*-knockdown seedlings infected by *Pst* CYR23, the fungal content in wheat tissues was significantly (*p* < 0.05) increased compared with that in the control plants. Relatively more abundant fungal biomass was identified in *TaATG8j-2s*-knockdown plants than that in the *TaATG8j-1s*–knockdown plants, which may have been due to the higher silencing efficiency of the second fragment than the first fragment (Figure 10).

## 3. Discussion

Autophagy is conserved among yeasts, animals and plants, and plants retain a majority of the autophagy machinery of yeast. In fact, several core protein families involved in autophagy have expanded in plants. As one of the essential autophagy-associated proteins, the expanded ATG8 family plays multifunctional roles in plants. In wheat, as a hexaploid (AABBDD) crop, nine ATG8-heterologous proteins have been reported [29]. In the present study, we identified and functionally characterized one of the ATG8 genes (*TaATG8j*) in wheat. Three copies of *TaATG8j* were located in the wheat genome. They shared high similarity with the other ATG8 members, which may suggest potential redundancy, or on the other hand, that many similar but different proteins or enzymes are required for the overall autophagy process.

During the wheat–*Pst* incompatible interaction, *TaATG8j* is specifically induced in the wheat tissue response to avirulent *Pst*, suggesting its potential involvement in the basal defense activated by avirulent rust fungus. Heterologous expression in fission yeast revealed a pro-cell-death function of *TaATG8j*. The silencing of *TaATG8j* resulted in reduced necrotic cell death caused by an avirulent *Pst* pathotype. During the wheat–*Pst* incompatible interaction, HR surrounding the infection sites with rapid and robust cell death is the main form of wheat resistance. The suppressed necrotic cell death indicated a prohibited HR in *TaATG8j*-knockdown plants, which led to the enhanced growth and development of *Pst*. It appears that *TaATG8j* functions positively to promote cell death during HR to defend against biotrophic pathogen attack. However, the overexpression of *TaATG8j* in *N. benthamiana* delayed cell death caused by BAX, exhibiting a pro-survival role. These results indicate that *TaATG8j* may play different roles under different conditions. In fact, there is still controversy about autophagic activities in cell death. Autophagy has been reported to be involved in both cell survival and cell death, which therefore to restrict or promotes PCD at the site of pathogen infection. In plants’ defensive response against biotrophic or necrotrophic fungi, PR proteins control pathogen invasion [9]. In stress conditions, autophagy triggers the cell survival (antideath) mechanism to cope with the adverse situation [33,34]. In contrast, in certain physiological or developmental conditions, autophagy is considered a nonapoptotic (autophagic or type II) form of cell death (pro-death) [35,36]. When there is limited nutrition, autophagy promotes or restricts PCD in specific pathological and developmental situations in eukaryotic cells, maintaining the adaptive and homeostatic balance [37]. The dual role of the plant immune system diagnoses pathogen-associated molecular patterns (PAMPs) by PAMP-triggered immunity (PTI) and evolves resistance proteins, which recognize the pathogen effector proteins and induces effector-triggered immunity (ETI). However, in most of the cases, PTI and ETI are related to PCD at the site of microbial invasion, which is considered HR [38].

Effector-triggered immunity is the main resistance mode of wheat to the biotrophic stripe rust fungus. As the typical characteristics of ETI, we measured HR, number of branches, hyphal length, and necrotic areas to determine the capability of *Pst* to form colonies in inoculated wheat tissue. The accumulation of PR proteins significantly decreased when TaPR1, TaPR2 and TaSOD primers were used in *TaATG8j*-knockdown plants (Figure 8), supporting a positive role of *TaATG8j* in the activation of the plant resistance response. For further confirmation of the function of *TaATG8j*, we assayed the fungal biomass in both BSMV:TaATG8j-1s- and BSMV:TaATG8j-2s-knockdown plants compared with the control (BSMV:γ) following infection with the avirulent *Pst* pathotype CYR 23. The *Pst* biomass in the knockdown plants was significantly increased (Figure 10), suggesting that TaATG8j proteins contribute to resistance to *Pst* by limiting *Pst* growth.

Determination of the subcellular localization may provide information about the functions of *TaATG8j*. In *A. thaliana*, ATG8 proteins penetrate the autophagosomes and vacuoles and then degrade or become attached to the outer membrane of the vacuoles, followed by removal from the outside membrane [18]. ATG8 proteins are scattered throughout a preautophagosomal structure under basal and low-nitrogen conditions [39]. ATG8 proteins participate in both autophagy and cytoplasm-to-vacuole transportation [20]. In wheat, four types of ATG8 proteins are distributed in punctate, possibly autophagosomal structures, suggesting that they may be recruited to autophagic membranes and then participate in autophagy [29]. In the present study, TaATG8j proteins were found also to be randomly distributed throughout the cytoplasm. The localization of TaATG8j proteins in the cytoplasm may suggest its function in autophagic membrane biogenesis. Whether the decreased defense in *TaATG8j*-knockdown plants is due to the altered autophagy activity needs to be further determined.

## 4. Materials and Methods

### 4.1. Cloning and Sequence Analyses of TaATG8j

Based on the expressed sequence tag (EST) sequence (Accession No. GR302394.1) from the cDNA library of a compatible wheat–*Pst* interaction [40], the primers TaATG8j-PB-F and TaATG8j-PB-R (Table S1) were used to clone the open reading frame (ORF) of *TaATG8j*. The amino acid sequence and conserved domains of TaATG8j were analyzed on website for protein translation (Available online: http://insilico.ehu.es/translate/) and NCBI (Available online: https://www.ncbi.nlm.nih.gov/Structure/cdd/wrpsb.cgi/), respectively. Multi-sequence alignment was conducted using DNAMAN 6.0 software (Lynnon Biosoft, San Ramon, CA, USA). Phylogenetic analysis of TaATG8j protein and other proteins was carried out using the neighbor-joining method with 1000 replicate bootstrap values, using the MEGA7 software (Available online: https://www.megasoftware.net/) (Figure 1 and Figure 2).

### 4.2. Plant and Fungal Materials

The wheat variety Suwon 11, *N. benthamiana* and *Pst* pathotypes CYR 31 (virulent) and CYR23 (avirulent) were collected from State Key Laboratory, Northwest A&F University, Yangling, China, and were used in this study. Su11, carrying the stripe rust resistance gene *YrSu*, is resistant to CYR23 but susceptible to CYR31 [41]. Wheat plants were grown and inoculated with the *Pst* pathotypes following the methods as described by Kang and Li [42]. The first leaves of wheat at the two-leaf stage were artificially inoculated separately with the fresh urediniospores of the *Pst* pathotypes CYR23 and CYR31, whereas the mock (control plant) was treated with sterile water. After inoculation, the wheat seedlings were incubated in a dark chamber for 24–36 h at 100% relative humidity and 15 °C temperature under a 16-h photoperiod with florescent white light. For RNA extraction, inoculated wheat leaves were sampled at 0, 6, 12, 24, 48, and 120 h post-inoculation (hpi). At each time point, sampled leaves were immediately submerged in liquid nitrogen and preserved at −80 °C prior to RNA extraction. For each time point, three biological replications were performed.

### 4.3. Extraction of RNA, cDNA Synthesis and qRT-PCR Analysis

RNA was extracted from the collected wheat leaves using the TRIzol reagent method (Invitrogen, Carlsbad, CA, USA), according to the guidelines of the manufacturer. The quality of RNA was determined by gel electrophoresis. Additionally, the RNA concentration was determined using a NanoDrop™1000 spectrophotometer (Thermo Fisher Scientific, Waltham, MA, USA). The cDNA was produced from 2 µg of total RNA with oligo (dt)18 primers using the RevertAid First Stand cDNA Synthesis kit (Thermo Fisher Scientific, Waltham, MA, USA; Available online: www.thermofisher.com/order/catalog/product/K1621) following the manufacturer’s instructions. On the basis of the subgenomic alignment to assess *Pst* expression, we designed specific primers from subgenomes 2A, 2B and 2D, and then qRT-PCR was performed (Figure 3). The expression of *TaATG8j-2AS*, *TaATG8j-2BS* and *TaATG8j-2DS* in relation to the compatible and incompatible interactions between wheat and *Pst* was quantified using qRT-PCR performed on a CFX Connect™ Real-time PCR Detection System (Singapore). The relative expression was controlled using the wheat elongation factor gene *TaEF-1α* (GenBank accession no. Q03033) and was quantified using the comparative 2^−ΔΔ*C*t^ method [43]. All reactions were performed in triplicate and with three biological replications. The primers used for qRT-PCR are listed in Table S1.

### 4.4. Subcellular Localization and Immunoblotting of GFP-TaATG8j

To investigate the subcellular localization of *TaATG8j*, the ORF of *TaATG8j* was sub cloned into the P^BinGFP2^ vector using the specific primers TaATG8j-PB-F and TaATG8j-PB-R. Furthermore, the common primers P^BinGFP2^-F and P^BinGFP2^-R were used to confirm the ligation of the ORF into the P^BinGFP2^-GFP vector, which was introduced into the *Agrobacterium tumefaciens* strain GV3101 by electroporation. When the OD_600_ of the culture medium reached 0.7–0.8 (4–5 weeks), we infiltrated the culture into *N. benthamiana* leaves. Infiltrated plants were incubated in a growth chamber under a 16-h/8-h photoperiod at 22 °C. Two days post-infiltration, leaf tissues were sampled for microscopic study using DAPI (concentration of 5 µg·mL^−1^) to detect whether GFP-TaATG8j was in nuclei. Additionally, hypertonic solution (0.8 M D-mannitol, MW: 182.17) was used to identify the specific position of GFP-TaATG8j. The GFP level of the empty vector and *TaATG8j* was observed under a fluorescence microscope (Olympus BX-53F, Olympus Corporation, Tokyo, Japan).

For Western blotting, leaves of *N. benthamiana* carrying eGFP-TaATG8j-PB-3101 were sampled and ground in liquid nitrogen. The total protein was extracted using a protein extraction kit (Solarbio, Beijing Solarbio Science and Technology Co. Ltd., Beijing, China) following the manufacturer’s guidelines. Western blotting was performed using 1× SDS-PAGE. Proteins were then transferred onto cellulose blotting membranes (pore size 0.45 mm, Bio-Rad, Hercule, CA, USA), which were incubated in blocking buffer (5% BD-Difco skim milk in 1× TBS and 0.05% Tween 20) for 2 h. The membranes were then incubated with mouse primary antibody (anti-eGFP antibody, Sigma-Aldrich, Shanghai, China) at 1:1000 dilutions to detect the eGFP fusion protein. Moreover, secondary antibody (anti-mouse antibody at 1:5000 dilution, Sigma-Aldrich Co.) and chemiluminescence substrate were used to visualize proteins (Sigma, Tokyo, Japan).

### 4.5. Agrobacterium-Mediated Transient Expression of TaATG8j in N. benthamiana

The coding region of *TaATG8j* was amplified with the specific primers TaATG8j-ClaI-F and TaATG8j-SalI-R (Table S1) and cloned via *Cla*I and *Sal*I into the potato virus X (PVX) vector PGR106, resulting in the recombinant PVX-TaATG8j. The reconstructed vectors PVX-Empty Vector (EV), PVX-Avr1b, PVX-BAX and recombinant PVX-TaATG8j were transformed separately into *A. tumefaciens* (GV3101) via electroporation. The transformed *A. tumefaciens* strains carrying PVX-EV, PVX-TaATG8j, PVX-BAX or PVX-Avr1b were cultured in LB medium with kanamycin (30 μg mL^−1^) and rifampicin (30 μg·mL^−1^) at 28 °C for 24–48 h. During the log phase, cells were collected by centrifugation at room temperature, washed 2–3 times with 10 mM MgCl_2_ and suspended to an OD_600_ of 0.2–0.3 with infiltration medium (10 mM·MgCl_2_). The suspensions were kept in darkness at room temperature for 3–4 h prior to infiltration. *A. tumefaciens* carrying PVX-EV, PVX-Avr1b, or PVX-TaATG8j were infiltrated into both sides of *N. benthamiana* leaves with a syringe without a needle. Twenty-four hours later, an *A. tumefaciens* strain carrying PVX-BAX was injected into the same position in one part of the leaf and was photographed 5 and 6 days after infiltration. Three biological replications were performed, and for each replicate, four *N. benthamiana* leaves were tested.

### 4.6. Overexpression of TaATG8j in Yeast

The coding sequence of *TaATG8j* was amplified using primers TaATG8j-*Sal* I-F and TaATG8j-*Sma* I-R and cloned into the *Sal*I- and *Sma*I-digested pREP3X vector. The reconstructed pREP3X_BAX, pREP3X and recombinant pREP3X_TaATG8j were transformed into fission yeast (*S. pombe*) by electroporation; 5 μg mL^−1^ thiamine (VB) was used to repress the nmt promoter in the pREP3x vector. The positively transformed yeast cells were incubated for 34 h in fresh liquid SD (-Leu) with or without thiamine with a starting optical density at OD_600_: 0.2. The fission yeast cells were then sampled at 14, 18, 22, 26, 30 and 34 h post-incubation. The dead cells were stained with trypan blue at a concentration of 10 µM [44] and then counted using a hemocytometer under an OLYMPUS BX-53F microscope. The dead yeast cells appeared blue, and the percentage of dead yeast cells to total yeast cells was determined in at least 10 fields of view. The total alive yeast cells were also counted with and without using VB.

### 4.7. BSMV-Mediated Silencing of TaATG8j in Wheat–Pst Interactions

The cDNA sequence of *TaATG8j* was aligned with the *T. aestivum* cv. Chinese Spring (CS) genome using the service provided by the International Wheat Genome Sequencing Consortium (Available online: http://wheat-urgi.versailles.inra.fr/Seq-Repository/BLAST/). Two fragments (105 bp from the coding region and 101 bp from the coding and 3′ untranslated regions) exhibiting the highest polymorphism within the gene family and the lowest sequence similarity to other genes were chosen to reconstruct gRNA-based derivative plasmids. The two chosen fragments used for silencing were amplified with the specific primers TaATG8j-PacI-F1, TaATG8j-NotI-R1, TaATG8j-PacI-F2 and TaATG8j-NotI-R2 (Supplementary Table S1) and sub-cloned into γ-RNA of barley stripe mosaic virus (BSMV):RNAs via the *Not*I and *Pac*I restriction sites to construct the recombinant BSMV:TaATG8j-1s and BSMV:TaATG8j-2s plasmid vectors. The silencing construct plasmids were linearized, and then BSMV:RNA was prepared using an in vitro RNA transcription kit (mMESSAGEmMACHINE; Ambion). For viral inoculation, the transcripts were diluted four times, including BSMV:RNA α, β, γ, γ-TaPDS, TaATG8j-1s and TaATG8j-2s. Transcript (RNA plasmid) was mixed with FES buffer [45], and then the mixtures (each leaf contained α:0.5, β:0.5, γ or γ-TaPDS or TaATG8j-1s or TaATG8j-2s:0.5 µl and FES buffer:9.0 µL) were inoculated on the apical side of the second leaves at the two-leaf stage by mildly rubbing the leaf surface using a gloved finger [46]. The leaves were incubated in the dark at a high humidity at 22–24 °C for 24 h. Subsequently, the virus-inoculated seedlings were shifted into a 23 °C growth chamber, and the virus symptoms were observed at regular intervals. BSMV:TaPDS was used as a positive control for the infection of BSMV. At 13 days post inoculation (dpi), when apparent viral symptoms appeared, the fourth leaf of each seedling was inoculated with fresh urediniospores of CYR23 for an incompatible interaction or CYR31 for a compatible interaction, and the seedlings were incubated at 16 °C with high relative humidity. The *Pst*-inoculated wheat leaves were collected at 0, 24, 48, 72 and 120 hpi for RNA extraction and histological observation. At 15 dpi, disease symptoms first appeared, and at 18 dpi the disease phenotype was photographed. Leaves were collected (only inoculated with CYR23) for biomass assay. For each assay, three replications were performed, and there were 150 seedlings for each replication.

### 4.8. Expression of TaATG8j and Pathogenesis-Related Protein (PR) Genes in TaATG8j-Knockdown Plants

The relative silencing efficiency of *TaATG8j* in wheat leaves inoculated with BSMV:TaATG8j-1s and BSMV:TaATG8j-2s was analyzed in comparison to BSMV: γ using qRT-PCR at 0, 24, 48, 72 and 120 hpi after *Pst* inoculation. The relative transcription levels of the wheat-pathogenicity-related (*PR*) protein genes *TaSOD* (KC158224.1), *TaPR1* (AAK60565) and *TaPR2* (DQ090946) in the *TaATG8j*-knockdown plants were determined by qRT-PCR.

### 4.9. Histological Study of Fungal Growth in TaATG8j-Knockdown Plants

For histological observation, wheat leaves were decolorized in acetic acid/absolute ethanol (1:1 *v*/*v*) and then fixed and cleared in trichloroacetaldehyde hydrate until the leaves were translucent. The cleared leaf segments were examined with a fluorescence microscope (OLYMPUS BX-53F). The autofluorescence of mesophyll cells at the infected site was observed and indicated a necrotic area. The fungal structure was stained with wheat germ agglutinin (WGA) conjugated to Alexa 488 (Invitrogen, Carlsbad, CA, USA) [47]. Only the infection in which an appressorium formed over the stoma was considered an actual penetration with the formation of infection hyphae, and the necrotic areas, haustorial mother cells, hyphal branch and hyphal length were examined. At least 50 infection sites were measured in each treatment. The hyphal length, number of hyphal branches and necrotic areas were calculated using DP2-TWAIN/DP2-BSW software (Olympus Corp, Tokyo, Japan). Error bars indicate the variation among the treatments. The statistical analysis was carried out using the Tukey test (*p* < 0.05).

### 4.10. Fungal Biomass Assay in TaATG8j-Knockdown Plants

Plant leaf samples were collected at 18 days post-inoculation with *Pst* CYR23 and the genomic DNA was extracted using the Plant Genomic Extraction Kit (TIANGEN, Beijing, China). The quantification of the *Pst* biomass was performed by qRT-PCR. The genomic DNA of Su11 leaves and urediniospores of *Pst* CYR23 were diluted in a gradient and used to prepare standard curves. Wheat *TaEF-1α* and the constitutively expressed *Pst* elongation factor *Pst*-EF [48] were used to quantify the wheat and *Pst* in the *Pst*-infected leaves of the BSMV:00- or BSMV:TaATG8j-inoculated plants. The two standard curves were used to perform the relative quantification of *Pst* and wheat genomic DNA.

### 4.11. Statistical Analyses

Mean values and standard errors were measured using Microsoft Excel, and statistical significance levels were assessed using the SPSS software (SPSS Inc. Chicago, IL, USA).

## 5. Conclusions

In conclusion, our study revealed the different roles of *TaATG8j* in cell death regulation in response to different stimuli. More importantly, our findings suggest that *TaATG8j* functions as a positive regulator of cell death in HR to promote defense against *Pst*. Further research is needed to explore the detailed regulatory mechanism of *TaATG8j* in autophagy and pathogen resistance regulation.

## Figures and Tables

**Table 1:** Three copies of *TaATG8j* in the subgenomes of wheat encoding the same TaATG8j protein isolated from the *Triticum aestivum* cv. Chinese Spring genomic database (Available online: http://plants.ensembl.org).

Gene	Chromosomal	cDNA	ORF	Protein	DNA	Identity (%) ^a^
	Location	Size (bp)	Size (bp)	Size (aa)	Size (bp)	Identity
*TaATG8j-2AS*	2AS	731	360	119	2438	99.3
*TaATG8j-2BS*	2BS	784	360	119	2746	98.4
*TaATG8j-2DS*	2DS	813	360	119	2810	98.6

^a^ The percentage of the identity was obtained by comparing the gene DNA sequences of Su11 with wheat genome cv. Chinese Spring.

## Supplementary Materials

The following are available online at http://www.mdpi.com/1422-0067/19/6/1666/s1.

## Abbreviations

ATG	autophagy-related gene
Cq	quantification cycle
DPI	days post-inoculation
hpi	hours post-inoculation
EST	expressed sequence tag
GFP	green fluorescent protein
HR	hypersensitive response
PCD	programmed cell death
PEG	polyethylene glycol
qRT-PCR	quantitative real-time PCR
*Pst*	*Puccinia striiformis* f. sp. *tritici*
VIGS	virus-induced gene silencing
