# Supplementary material for: Wheat Gene TaATG8j Contributes to Stripe Rust Resistance
Source: Int J Mol Sci. 2018 Jun 5;19(6):1666. doi: 10.3390/ijms19061666 (PMC6032272; doi:10.3390/ijms19061666)
Supplement: Supplementary file 1 [file ijms-19-01666-s001.pdf]

Supplementary Table S1. Primer used for TaATG8j analysis

Name of Primers	Sequences (5′→3′)	Amplified length (bp) /type of Oligonucleotides	Purpose
M13-F	TGTAAAACGACGGCCAGT	General primer	Checking
M13-R	CAGGAAACAGCTATGACC		
PVX-F	CAATCACAGTGTTGGCTTGC	General primer	Checking
PVX-R	GACCCTATGGGCTGTGTTG		
pREP3x-S	CAGCGAAACTAAAAACCG	General primer	Checking
pREP3x-AS	TCTAAAAGCGAAAAACAAAAT		
¦Ã-as-seq-F	AAAGTGAGGTTAACGCAATACG	General primer	Checking
¦Ã-as-seq-R	TCAGGCATCGTTTTCAAGTT		
TaATG8j-ClaI-F	ggcatcgat ATGGCCAAGACTTGCTTCA	360	Expression in Tobacco
TaATG8j-SalI-R	catgtcgacTTAGGCAGAGCCGAAAGT		
TaATG8j -SalI-F	catgtcgac ATGGCCAAGACTTGCTTCA	360	Expression in Yeast
TaATG8j -SmaI-R	gtacccggg TTAGGCAGAGCCGAAAGT		
TaATG8j-PB-F	ctgtacaagggtacccccggg ATGGCCAAGACTTGCTTCAA	357	Sub-cellular localization
TaATG8j-PB-R:	tctagttcatctagaggatcctta GGCAGAGCCGAAAGTGTT		
pBinGFP2-F	AAGACCCCAACGAGAAGC	General primer	Checking
pBinGFP2-R	GAACCCTAATTCCCTTATCTG		
TaATG8j-PacI-F1	atattaattaaCTACGTGGTGCGGAAGAGG	104	VIGS-1
TaATG8j-NotI-R1	tatgcggccgcTCATAGATGGCTGACATCAAC		
TaATG8j-PacI-F2	atattaattaaTCTGCCTAATCCATGTGCG	101	VIGS-2
TaATG8j-NotI-R2	tatgcggccgcTGTGAGCAATCCACCAGCA		
TaATG8j-2AS: QT-F	CCATCTTCGTCTTTGTGAATAGC	177	
TaATG8j-2AS: QT-R	GCAACCTGAACATCCATTTATTT		
TaATG8j-2BS: QT-F	AATCTCGTCCCAGGCTGCG	167	
TaATG8j-2BS: QT-R	GAGGTCGGCAGGGACAAGG		
TaATG8j-2DS: QT-F	AACACTTTCGGCTCCGC	199	
TaATG8j-2DS: QT-R	TCTTATCTATTACTCTCGTCTGGTT		
TaEF-qRT-F	TGGTGTCATCAAGCCTGGTATGGT	General primer	
TaEF-qRT-R	ACTCATGGTGCATCTCAACGGACT		
biomass-PstEF-F	TTCGCCGTCCGTGATATGAGACAA	General primer	Biomass assay
biomass-PstEF-R	ATGCGTATCATGGTGGTGGAGTGA		
biomass-WEF-F	TGACCAGATCAACGAGCC	General primer	
biomass-WEF-R	CTCCAGGAGAGACTCATG		
TaPR1-S	GAGAATGCAGACGCCCAAGC	General primer	Pathogenesis Related
TaPR1-AS	CTGGAGCTTGCAGTCGTTGATC		
TaPR2-S	AGGATGTTGCTTCCATGTTTGCCG	General primer	
TaPR2-AS	AAGTAGATGCGCATGCCGTTGATG		
TaSOD-S	CCGAGGTCTGGAACCATCAC	General primer	
TaSOD-AS	AGCCGAAATCCTTCTCGATCT		

*The Uppercase letters indicate bases that match the initial template. Lowercase letters which are underlined in 5′ extensions that indicates the restriction sites.

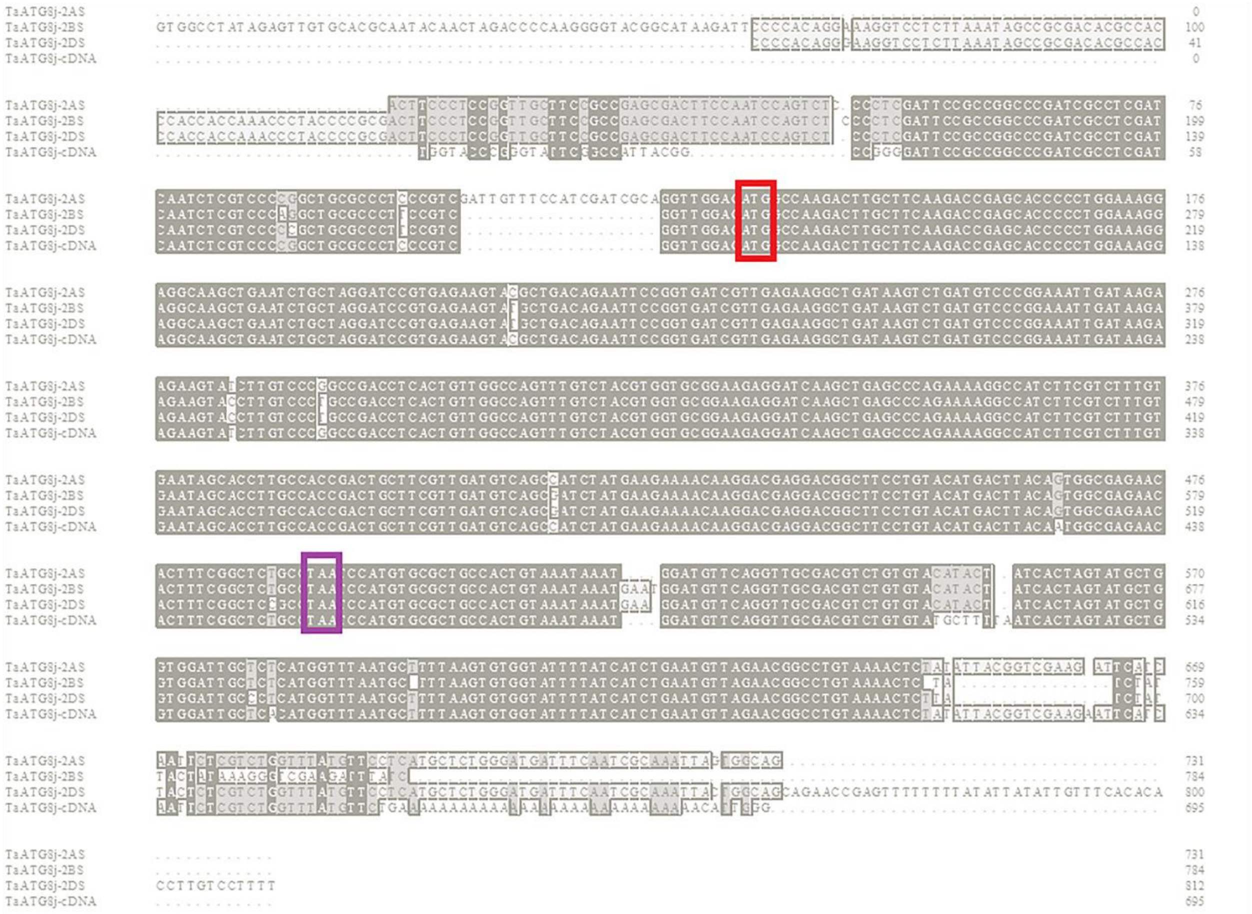

Figure S1**.** Alignment of the three subgenomic copies of *TaATG8j*, *TaATG8j* cDNA, the full-length cDNA sequence, was aligned from the wheat cultivar cv. Su11. The other cDNA sequences (*TaATG8j-2AS*, *TaATG8j-2BS* and *TaATG8j-2DS*) of *TaATG8j* were obtained from the Chinese Spring URGI genome database. The purple and red boxes indicate the start codon (ATG) and stop codon (TAA), respectively. Identical nucleotides are in black.

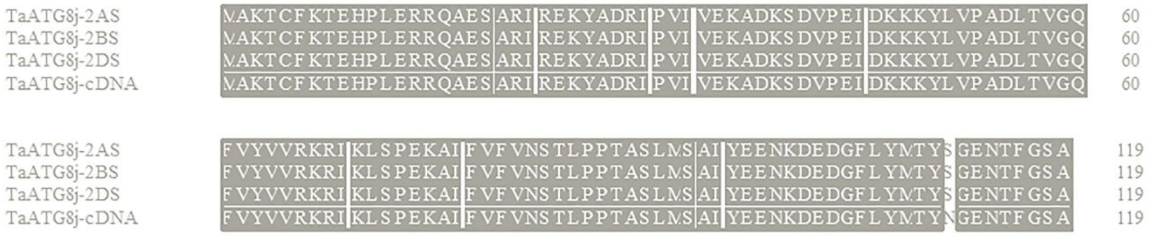

Figure S2. Multiple alignment of the TaATG8j proteins of wheat cv. Su 11 with three copies of the Chinese Spring Wheat Genomic Database. Identical amino acid residues are in black.

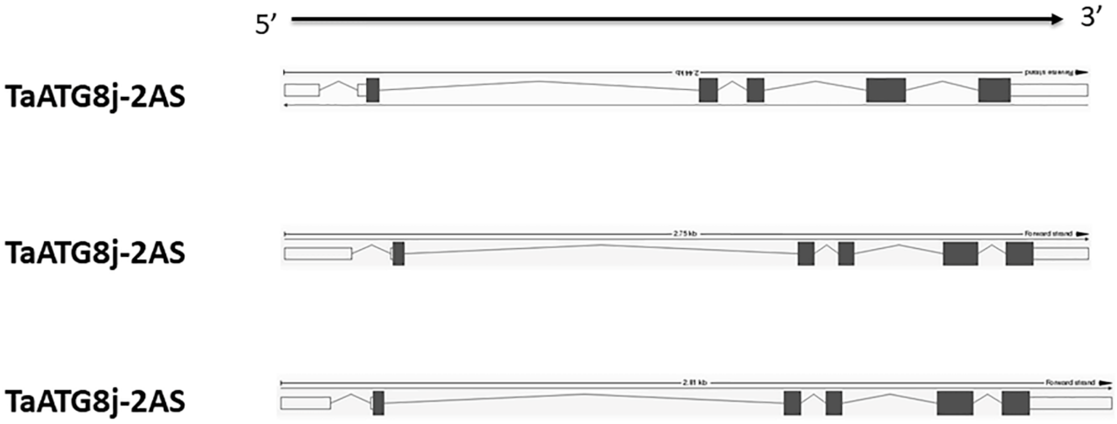

Figure S3. Diagram showing the three subgenomic copies of *TaATG8j* from the wheat cv. Chinese Spring URGI genome database. Solid rectangles indicate exons, and zigzags indicate introns. The lengths of the three copies were *TaATG8j- 2AS*, 2.44 kb; *TaATG8j-2BS*, 2.75 kb; and *TaATG8j-2DS*, 2.81 kb.

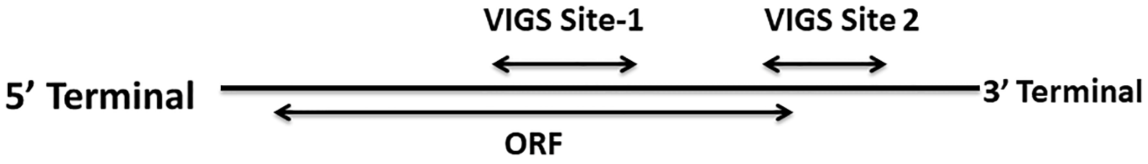

Figure S4. Virus-induced gene silencing fragments. Two fragments were considered from the open reading frame (ORF) and the ORF plus the 3’ noncoding region of the *TaATG8j* gene for specific knockdown. ORF: open reading frame. VIGS Site -1: *TaATG8j* -1s. VIGS Site -2: *TaATG8j*-2s.
